# Anti-Phthisis Activities in Berkshire

**Published:** 1914-03-14

**Authors:** 


### ANTI-PHTHISIS ACTIVITIES IN BERKSHIRE.

The great majority of county councils and councils of county boroughs have already formulated schemes in connection with the fight against tuberculosis; such provision is now obligatory, and at the present time there are only six defaulters among the county councils and seven among the county boroughs. But this is far from meaning that all the other authorities have adequate schemes in working order. As a matter of fact it will be a long time before the word "adequate" can be applied to many of the schemes, and possibly several months will have elapsed before a large number are in working order.

The organisation of the anti-tuberculosis campaign in Berkshire is proceeding on satisfactory lines, although the complete scheme is somewhat slow in maturing. An important change has recently been rendered necessary owing to the appointment of the county tuberculosis officer, Dr. Norris, to a very responsible post under the Home Office. The vacancy thus created has just been filled by the election of Dr. A. Richmond to the post of tuberculosis officer for the county. Dr. Richmond leaves the enterprising county of the West Riding of Yorkshire, which has been the training school of quite a large number of men now holding good posts in the public health services, and he will, we have no doubt, find plenty of scope for his organising abilities in setting up an efficient anti-tuberculosis scheme in the home county.

The recommendations of the county council sub-committees as far as they have gone at present do not favour the initiation immediately of a complete scheme, which must involve a considerable burden on the rates, but suggest the continuance and development of the present "dispensary" scheme. The word "dispensary" in this connection needs a little explanation, for in Berkshire the scheme does not necessarily mean the provision of dispensary buildings at centres throughout the county, but for present purposes refers to an organisation consisting of a tuberculosis officer and staff with a central office. This so-called dispensary system is very shortly to be strengthened by the addition of two whole-time nurses, a clerk on the staff of the county medical officer of health, Dr. Gerard Taylor, and by the co-operation of nursing associations in the county. In a rural area, such as Berkshire, it is sufficiently obvious that visits to the homes must form a very important part of the tuberculosis officer's work, and that the gathering of patients and contacts at stated times at certain centres has many disadvantages and few advantages.

In the Metropolis the contrary holds good. In the country long distances will have to be traversed by invalids, and probably carriers' carts will often be the means of conveyance. In these unhygienic vehicles the spectacle of several consumptives coughing and spitting in a cramped and crowded space is not the sort of thing to be regarded with equanimity by a tuberculosis officer. When all is said and done it is undoubtedly in the homes of the consumptives that the disease must be attacked; the tuberculosis officer must of necessity visit the rooms inhabited by his patients in order to support with his advice and authority the recommendation of the visiting nurse, and the only practicable way to ensure the "catching of contacts" for examination purposes is to go after them. Thus a system of dispensaries in a rural county without fairly large towns would be an unnecessary if not a futile complication.

